# Anais Brasileiros de Dermatologia - Impact Factor and CiteScore for 2023^☆^

**DOI:** 10.1016/j.abd.2024.07.002

**Published:** 2024-08-06

**Authors:** Silvio Alencar Marques, Ana Maria Ferreira Roselino, Hiram Larangeira de Almeida Jr, Luciana Patrícia Fernandes Abbade

**Affiliations:** a Department of Infectology, Dermatology, Imaging Diagnosis and Radiotherapy, Universidade Estadual Paulista, Botucatu, SP, Brazil; b Department of Internal Medicine, Division of Dermatology, Faculdade de Medicina de Ribeirão Preto, Universidade de São Paulo, Ribeirão Preto, SP, Brazil; c Department of Dermatology, Universidade Federal de Pelotas, Pelotas, RS, Brazil; d Department of Dermatology, Universidade Católica de Pelotas, Pelotas, RS, Brazil

The *Anais Brasileiros de Dermatologia* (ABD) was published for the first time under the name *Annaes Brasileiros de Dermatologia e Syphilographia* in 1925 and has been published uninterruptedly since then.^1^ It was indexed in PubMed/Medline in 2009 and the first Impact Factor (IF), published by the Journal of Citation Reports in 2010, corresponded to 0.337. In the following years, the IF increased practically constantly. In 2023, the IF increased to 2.6, corresponding to the highest value in its historical series. And, coherently, the CiteScore for 2023 was 2.4, repeating the 2022 index. In common, even though they use different methodologies, they measure the number of citations received in 2023 by a certain journal, for articles published in the two immediately previous years (IF), or, published in the four immediately previous years (CiteScore). Both metrics, biases aside, reflect the status and scientific hierarchy of scientific journals in the measured period. The ABD evolved in terms of IF, from 50^th^ position out of 70 journals in Dermatology and related areas in 2022, to 34^th^ position out of 94 in 2023. As for CiteScore, it went from the 60^th^ position out of 133 in 2022, to the 71^st^ position out of 142 journals in 2023. Therefore, placing it in Quartile 2 in both metrics it will be of special importance for Capes/MEC (The Brazilian Federal Agency for Support and Evaluation/Ministry of Education) in defining Qualis/ABD in 2024/2025.

Below, for recognition and reflection, are the five articles with the highest rates of citations received in the IF/ABD/2023 composition: 1^st^ - Orofino-Costa R et al.,^2^ 2^nd^ - Bittner GC et al.,^3^ 3^rd^ - Marchioro HZ et al.,^4^ 4^th^ - Barros NM et al. ^5^ and 5^th^ - Seque MA et al.^6^

The previously reported numbers and the most cited topics (see references) suggest that ABD has been eclectic and is one of the few journals in general Dermatology to open generous space to infectious diseases, and this sets it apart.^7^, ^8^

Likewise, the previous numbers reported indicate that the minimum threshold to be considered is IF = 2.6 and reinforces our commitment to constant efforts aimed at improvement and advances. The importance of Brazilian and Latin American Dermatology requires us to do so.

## Financial support

None declared.

## Authors' contributions

Silvio Alencar Marques: Approval of the final version of the manuscript; drafting and editing of the manuscript.

Ana Maria Roselino: Approval of the final version of the manuscript; drafting and editing the manuscript.

Hiram Larangeira de Almeida Junior: Approval of the final version of the manuscript; drafting and editing of the manuscript.

Luciana P. Fernandes Abbade: Approval of the final version of the manuscript; drafting and editing of the manuscript.

## Conflicts of interest

None declared.
